# Loss of Endothelial Barrier in Marfan Mice (mgR/mgR) Results in Severe Inflammation after Adenoviral Gene Therapy

**DOI:** 10.1371/journal.pone.0148012

**Published:** 2016-02-03

**Authors:** Philipp Christian Seppelt, Simon Schwill, Alexander Weymann, Rawa Arif, Antje Weber, Marcin Zaradzki, Karsten Richter, Stephan Ensminger, Peter Nicholas Robinson, Andreas H. Wagner, Matthias Karck, Klaus Kallenbach

**Affiliations:** 1 Department of Cardiac Surgery, University Hospital Heidelberg, Heidelberg, Germany; 2 German Cancer Research Center, Division of Molecular Genetics, Heidelberg, Germany; 3 Heart and Diabetes Center NRW, Clinic for Thoracic and Cardiovascular Surgery, Bad Oeynhausen, Germany; 4 Institute for Medical Genetics and Human Genetics, Charité University Hospital, Berlin, Germany; 5 Max-Planck Institute for Molecular Genetics and Berlin-Brandenburg Center for Regenerative Therapies (BCRT), Berlin, Germany; 6 Institute of Physiology and Pathophysiology, University of Heidelberg, Heidelberg, Germany; Max-Delbrück Center for Molecular Medicine (MDC), GERMANY

## Abstract

**Objectives:**

Marfan syndrome is an autosomal dominant inherited disorder of connective tissue. The vascular complications of Marfan syndrome have the biggest impact on life expectancy. The aorta of Marfan patients reveals degradation of elastin layers caused by increased proteolytic activity of matrix metalloproteinases (MMPs). In this study we performed adenoviral gene transfer of human tissue inhibitor of matrix metalloproteinases-1 (hTIMP-1) in aortic grafts of fibrillin-1 deficient Marfan mice (mgR/mgR) in order to reduce elastolysis.

**Methods:**

We performed heterotopic infrarenal transplantation of the thoracic aorta in female mice (n = 7 per group). Before implantation, mgR/mgR and wild-type aortas (WT, C57BL/6) were transduced *ex vivo* with an adenoviral vector coding for human TIMP-1 (Ad.hTIMP-1) or β-galactosidase (Ad.β-Gal). As control mgR/mgR and wild-type aortas received no gene therapy. Thirty days after surgery, overexpression of the transgene was assessed by immunohistochemistry (IHC) and collagen *in situ* zymography. Histologic staining was performed to investigate inflammation, the neointimal index (NI), and elastin breaks. Endothelial barrier function of native not virus-exposed aortas was evaluated by perfusion of fluorescent albumin and examinations of virus-exposed tissue were performed by transmission electron microscopy (TEM).

**Results:**

IHC and ISZ revealed sufficient expression of the transgene. Severe cellular inflammation and intima hyperplasia were seen only in adenovirus treated mgR/mgR aortas (Ad.β-Gal, Ad.hTIMP-1 NI: 0.23; 0.43), but not in native and Ad.hTIMP-1 treated WT (NI: 0.01; 0.00). Compared to native mgR/mgR and Ad.hTIMP-1 treated WT aorta, the NI is highly significant greater in Ad.hTIMP-1 transduced mgR/mgR aorta (p = 0.001; p = 0.001). As expected, untreated Marfan grafts showed significant more elastolysis compared to WT (p = 0.001). However, elastolysis in Marfan aortas was not reduced by adenoviral overexpression of hTIMP-1 (compared to untreated Marfan aorta: Ad.hTIMP-1 p = 0.902; control Ad.β-Gal. p = 0.165). The virus-untreated and not transplanted mgR/mgR aorta revealed a significant increase of albumin diffusion through the endothelial barrier (p = 0.037). TEM analysis of adenovirus-exposed mgR/mgR aortas displayed disruption of the basement membrane and basolateral space.

**Conclusions:**

Murine Marfan aortic grafts developed severe inflammation after adenoviral contact. We demonstrated that fibrillin-1 deficiency is associated with relevant dysfunction of the endothelial barrier that enables adenovirus to induce vessel-harming inflammation. Endothelial dysfunction may play a pivotal role in the development of the vascular phenotype of Marfan syndrome.

## Introduction

In patients with Marfan Syndrome (MFS) mutation of the fibrillin-1 gene (*FBN1)* leads to defective extracellular microfibrils, resulting in divergence and instability of the entire connective tissue [1]. Although MFS is a systemic disorder affecting the skeletal system, eyes, dura, and pulmonary system, reduced life expectancy is primarily determined by cardiovascular manifestations [2]. On the molecular level, there is a distinctive fragmentation of elastic fiber networks (elastolysis) that results in increased aortic stiffness and pulse wave velocity as main contributors for aortic dilatation [3]. Thus, dilatation of the aortic root is characteristic for MFS and can lead to acute dissection of the aorta [4]. Today, prophylactic replacement of the dilated aortic root with preservation of the native aortic valve leads to excellent results, allowing an almost normal life expectancy [5, 6]. However, prevention of root dilatation by new therapeutic targets must be the ultimate therapeutic aim for patients with MFS.

Degradation of elastin fibers correlates with calcification of aortic media, recruitment of inflammatory cells and overexpression of matrix metalloproteinases (MMPs) [7], [8]. MMPs play a crucial role in degradation and modification of the extracellular matrix [9]. The most significant endogenous inhibitors of MMPs are tissue inhibitor of matrix metalloproteinases (TIMPs). TIMP-1 is a glycoprotein and represents a potent inhibitor of collagenases, gelatinases, and proteoglycanases [10, 11]. It inhibits the activity of a wide range of MMPs and has been successfully applied in disease models triggered by MMP-overexpression and hyperactivity [12, 13]. Forough et al. revealed increased elastin accumulation in the intima of rat carotids after local overexpression of TIMP-1 [14]. The fibrillin-1 deficient mouse (mgR/mgR) represents an accepted murine model of MFS with a strong genotype-phenotype correlation [15, 16]. The mgR/mgR mouse shows typical morphological features of MFS and die of spontaneous dissections of aortic root aneurysms [17]. Furthermore increased activity of MMP-2 and MMP-9 was detected in the aortic wall of Marfan mice and application of the non-specific MMP-inhibitor doxycycline has proved its potential to reduced progression of aneurysm formation in the mgR/mgR as well as in a different Marfan mouse model based on a cysteine substitution in fibrillin-1 (Fbn1C1039G/+) [16, 18]. Therefore, the mgR/mgR mouse model is well suited for studying new molecular strategies for the treatment of MFS.

In this work we report on the adenoviral mediated gene transfer of hTIMP-1 in mgR/mgR mice with the goal of reducing MMP triggered elastolysis and stabilizing the aortic wall. As study model we chose heterotopic transplantation of the thoracic aorta into an abdominal position, which allows a safe and sufficient *ex vivo* adenoviral transduction of the aortic vessel between harvest and transplantation. In contrast to systemic application of the adenovirus over a tail vein unknown side effects can be limited and the effect of the transgene overexpression can be observed directly by *ex vivo* transduction. Objectives of this work were first the proof of feasibility of adenoviral gene therapy directed against elastolysis in Marfan mice and second to study interactions of adenovirus and aortic wall in order to reveal feasible targets for future gene therapy.

## Methods

### Animals

Wildtype (WT) C57BL/6 and homozygous mgR/mgR Marfan mice (C57BL/6 genetic background) at the age of 6–9 weeks were used for this study. Because female mgR/mgR mice are more robust than male and have shown a longer mean survival only female mice were used for surgery. This study was carried out in strict accordance with the recommendations in the Guide for the Care and Use of Laboratory Animals of the National Institutes of Health. The protocol was approved by local administration (Regierungspräsidium Karlsruhe, permit number: T36/08, T 07/11 and G17/09). According to requirements by local administration and in order to reduce the size of the animal breeding age of operated mice ranged between 6 to 9 weeks. All efforts were made to minimize suffering. Heterozygous females (mgR/-) were matched with homozygous males (mgR/mgR) to generate adequate quantity of homozygous mice. Wildtype mice were generated out of the same breed by breeding heterozygous (mgR/-) mice. For genotyping tag DNA was isolated and analyzed by quantitative real-time polymerase chain reaction as described before [17].

### Adenoviral Vectors

Adenovirus was generated by Harald A. Fernandez (New York University Medical Center) and Klaus Kallenbach. For this project we used adenovirus type 5, which lacks the E1A region and is therefore replication incompetent [19],[20]. Adenovirus vectors coding for human TIMP-1 (Ad-CMVhTIMP-1) and β-galactosidase (Ad-CMVβ-Gal.) were produced, purified and filtrated in N52.E6 cells according to standard protocols as described previously [19, 20], [21]. Virus titer was determined by TCID (tissue-culture-infective-dose)-test in A549 cells and replication competence was ruled out by RCA (replication-competent-adenovirus)-test in A549 cells as well [22], [21]. Suitable virus titer was selected by determination of transduction efficiency in Ad.hTIMP-1 transduced HEK-293 cells and Ad.β-Gal. transduced WT aortas (not part of this work). Virus concentration of 5x10^9^ pfu/ml presented the best ratio of transgene expression and virus toxicity. The used Ad.hTIMP-1 has proofed its potential to transduce human smooth muscle cells in vitro with high efficiency [22].

### Cell Culture

N52.E6 cells for cultivation of adenovirus, A549 cells for TCID-test and HEK 293 cells for western blotting were stored at -80°C. For cultivation cells were transferred either in D-MEM (HEK 293 and A549) or in alpha MEM (for N52.E6 cells) concentrated with 10% fetal calf serum, 1% glutamine, 0,1mg/ml penicillin and 0,1mg/ml streptomycin (Invitrogen Life technologies Corp., Carlsbad, USA). Cell medium was seeded and incubated at 37°C with 95% humidity and 5% CO2. At regular intervals cell culture were checked until a confluent cell lawn was formatted. Then cell medium was removed and the cells were washed with 5ml PBS and incubated with 3ml trypsin (0,25%, Carl Roth GmbH, Karlsruhe, Germany) for 3min. Generated cell titer was count in a Neubauer chamber (Neubauer improved counting chamber, Carl Roth GmbH, Karlsruhe Germany).

### Surgery

Overall 14 female WT mice and 21 female mgR/mgR mice at the age of 6–9 weeks underwent heterotopic transplantation of the thoracic aorta into an infrarenal position. Donor mice of the same age were euthanatized with CO_2_ and the abdomen was opened by median thoracotomy. The left ventricle was punctured and 10 ml of cold saline solution (4°C, 0,9%, Braun AG, Melsungen, Germany) was applied systemically. The thoracic aorta was dissected carefully and removed en bloc. Depending on the experimental group the graft was now either first transduced (see below) or only flushed with cold saline before transplantation. Recipient mice were narcotized with isoflurane and the abdomen was opened by median laparotomy. The intestines were mobilized and stored on wet gauze. The infrarenal aorta was then separated carefully from the inferior vena cava and para-aortic tissue. Before the aorta was cut transversely, vessel clips were set distal to the left renal artery and close to the bifurcation. The previously prepared donor aorta was then implanted with each 5 to 6 simple interrupted stitches by the end-to-end technique (Prolene 11–0, Nylon black, S&T AG, Neuhausen, Switzerland). To prevent distal ischemia the lower limbs were consistently cooled with cold saline solution. After finishing the anastomoses clips were removed and bleeding control was started. Thereafter, the intestines were repositioned and the abdomen and skin were closed by continuous stitches. Neither systemic nor local anticoagulation was used perioperatively. The postoperative protocol included mobilization and massage of lower limbs to guarantee distal perfusion and analgesia every eight hours for three consecutive days (Buprenorphine, 0.05mg / kg body weight). Grafts were harvested 30 days post-surgery.

### Adenoviral Transduction

For adenoviral transduction, the ascending aorta was cannulated with a blunt cannula and two knots were set to secure positioning within the aorta. The aorta was clipped distally and inoculum injected for incubation. In addition the aorta was placed within the virus solution for 5 minutes to obtain virus transduction from the adventitial side as well. In order to increase transduction efficiency, adenovirus was dissolved in Poloxamer 407 (21%, BASF, Ludwigshafen, Germany). After 30 minutes in 37°C incubation solution (Dulbecco's Modified Eagle's Medium, DMEM 2%, 10% fetal calf serum, FCS), the clip was removed and the aorta was flushed several times with cold saline solution for complete removal of virus solution. Aortic grafts of mgR/mgR mice (n = 7) and WT mice (n = 7) were treated with 1 ml adenoviral vector solution coding for hTIMP-1 (Ad.hTIMP-1, 5x10^9^ pfu/ml). Furthermore, mgR/mgR mice (n = 7) were treated by vector coding for β-galactosidase (1 ml, Ad.β-Gal., 5x10^9^ pfu/ml) to evaluate transfection. Finally, mgR/mgR (n = 7) and WT mice (n = 7) did not receive any perioperative adenoviral treatment and were simply flushed with cold saline and incubated after the same protocol.

### Western Blotting

Western blotting was accomplished to show overexpression of human TIMP-1 protein after transduction with Ad-hTIMP-1. Therefore HEK-293 cells were transduced (60min, 5x10^9^ pfu/ml) and cell supernatant was centrifugalized after standard protocols for protein isolation. Protein concentration was measured photometrically by Bradford protein assay (Dye-Reagent-Concentrate, Bio-Rad Laboratories GmbH, München, Germany; Hitach U200, Hitachi Ltd., Tokyo, Japan) Proteins were separated by SDS (sodium dodecyl sulfate) polyacrylamide gel electrophoresis (ready-made gels, Invitrogen, Life technologies Corp., Carlsbad, USA; broad range protein marker, New England Biolabs, Ipswich, USA) and transferred by western blotting on a polyvinylidene fluoride membrane at 50 volt using a standard blot chamber (Invitrogen Life Technologies Corp., Carlsbad, USA). After overnight blocking in phosphate buffered saline (PBS solution) blot membrane was incubated with primary (three hours, 1:1000 in PBS, human TIMP-1 antibody, MAB 3301, Merck Millipore, Darmstadt, German) and secondary antibody (one hour, 1:5000 in PBS, horse radish peroxidase linked, AP 308P, Merck Millipore, Darmstadt, Germany). Finally, secondary antibody was detected to determine protein lanes (Fujifilm film cassette, Tokyo, Japan; ECL Plus Western Blotting Detection reagent, GE Healthcare Amershan, USA).

### Human TIMP-1 Immunohistochemistry

Immunohistochemical staining was performed on aortic grafts 30 days post transplantation to verify overexpression of human TIMP-1 after transduction of Ad.h-TIMP-1 prior to transplantation. Monoclonal murine antibody against human TIMP-1 was applied to aortic cryo-sections (human TIMP-1 antibody, MAB3301, Merck Millipore, Darmstadt, Germany) for 30 min at a dilution of 1:200 and visualized by secondary antibody labeled with horseradish peroxidase according to the manufacturer’s protocols (PK-2200, SP-2001 und Sk-4100, Vector Laboratories Inc., Burlingame, USA). Evaluation was performed by bright-field microscopy (Nikon Ni-E, Nikon Imaging Center, Nikon Imaging Software 4.6, Nikon, Tokyo, Japan).

### F4/80 Monocytes Fluorescence Immunohistochemistry

In order to outline cellular inflammation 30 days post transplantation immunofluorescence staining of frozen tissue sections was performed according to standard protocols. Rat anti-mouse F4/80 antibody against monocytes (T-2006, 1:200 dilution) was obtained from Dianova (Hamburg, Germany) and goat anti-human IgG-Cy3 was obtained from Sigma-Aldrich as secondary antibody (Taufkirchen, Germany). Cell nuclei were visualized by counterstaining with DAPI and fluorescence intensity was recorded using an Olympus IX81 confocal microscope (Olympus, Japan). Qualitative image analyses were performed using the Olympus Xcellence software (Olympus, Japan).

### Albumin Perfusion Test

In order to verify endothelial integrity, 6 native, not virus-exposed and not transplanted mgR/mgR aortas and 6 native, not virus-exposed and not transplanted WT aortas were perfused with fluorochrome labeled albumin (100μg/ml albumin from bovine serum, Alexa Fluor^®^ 594 conjugate Molecular Probes, Inc. Eugene, USA diluted in PBS) and after 30 minutes of incubation flushed accurately with cold saline. For statistical evaluation aortas were cut, cryofixed and analyzed in 200x magnification by fluorescent microscopy (Texas Red, absorptions maxima of 586nm and fluorescence emissions maxima of 605nm, Nikon Ni-E, Nikon Imaging Center Heidelberg, Nikon, Tokyo, Japan). The aortic media was defined as region of interest (ROI) and was selected semi-automatically by imaging software (Nikon Imaging Software 4.6, Nikon, Tokyo, Japan). To rule out systemic evaluation errors, in total 9 photos of each aortic media were taken in different parts of the aorta and mean of relative brightness was calculated for each media.

### Collagen *In Situ* Zymography

Activity of MMP-1, 2, 3, 9, and 13 within the tissue was visualized by collagen *in situ* zymography. Thirty days after transplantation grafts were harvested and cryofixed slides (5mm cut) of the aorta were covered and incubated with fluorescent proteinase substrate (dq-collagen, EnzCheck D-12060, Molecular Probes, Invitrogen, Carlsbad, USA) for 48h according to manufacturer’s instructions. Prior and after incubation pictures were taken with identical camera settings and matched for analysis. Differences in light emission as the result of collagen substrate conversion were analyzed by fluorescent microscopy. Dq-collagen has absorptions maxima of 495nm and fluorescence emissions maxima of 515nm.

### Microscopic Examination

Mice were euthanized with CO_2_ for graft examination 30 days post-surgery. Before graft explantation the heart was punctured and cold saline (4°C) was perfused systemically. For histologic examination aortas were cryofixed (Tissue-Tek^®^ O.C.T^™^ Compound, Sakura Finetek, Netherlands) and cut in 5-mm frozen sections. The graft was divided into 4 parts A to D (6–9 slices per part) to ensure a continuous evaluation. To analyze intimal hyperplasia, elastin breaks and inflammation Elastica van Gieson and Hematoxylin-Eosin staining was performed. Pictures were taken at 100x and 400x magnification (Nikon Eclipse 90i; Nikon Imaging Center, Heidelberg, Germany) and assessed by common imaging software (Image Pro Plus 4.5, Medi Cybernetics, San Diego, USA). Elastin breaks of the media were counted at 400x and neointimal index (NI, intima / (intima + lumen), %) was measured at 100x magnification. Area of the intima as region of interest (ROI) was defined semi-automatically using imaging software.

### Transmission Electron Microscopy

The specific structure of Marfan and WT vessels after adenovirus transduction was displayed by transmission electron microscopy. Two WT and two mgR/mgR thoracic aortas were harvested and transfected with Ad.hTIMP-1 as described above. After 7 and 30 minutes of incubation each one WT and one Marfan aorta was flushed intensively with cold saline and perfused with fixation solution (2% formaldehyde, 2% glutaraldehyde, 2mM MgCl_2_ in 66 mM cacodlyate-buffer, pH 7.2). After placing the aortas in fixation solution, tissue was cut in 0.5mm thick slices. Immersion-fixation was continued over night at 4°C in EM-fix. Tissue was post-fixed in buffered OsO4, dehydrated by ethanol of increasing concentration (50%, 75%, 100%) an embedded in epoxide (11.5 g Glycidether, 6g DDSA, 7.5g NMA, 850 μl BDMA, all from Sigma, St. Louis, Missouri, USA). Sections were cut at nominal thickness 60 nm, post-stained by uranyl and lead and observed with a Zeiss EM 910 at 80 kV (Zeiss, Jena, Germany).

### Statistical Analysis

All statistical evaluation was performed using SPSS (IBM SPSS Statistics 19; IBM Corporation, Armonk, NY, USA). If the data were normally distributed, a T-test was applied, otherwise the non-parametric Mann-Whitney U-test was performed. Statistical significance was accepted at p≤0.05, and results of p≤0.001 were described as highly significant. All photos were randomized and blinded before independent evaluation by two different researchers. For each aortic graft numbers of elastin breaks were counted and NI was measured in four different pictures, each of different aortic slides to calculate means. For Evaluation of Albumin perfusion test 9 photos of each media were taken in different parts of the aorta and mean relative brightness was ascertained in a blinded fashion.

## Results

### Transduction of Ad.hTIMP-1 Results in Overexpression of hTIMP-1 and Consecutive Reduced MMP Activity

**Fig 1:**
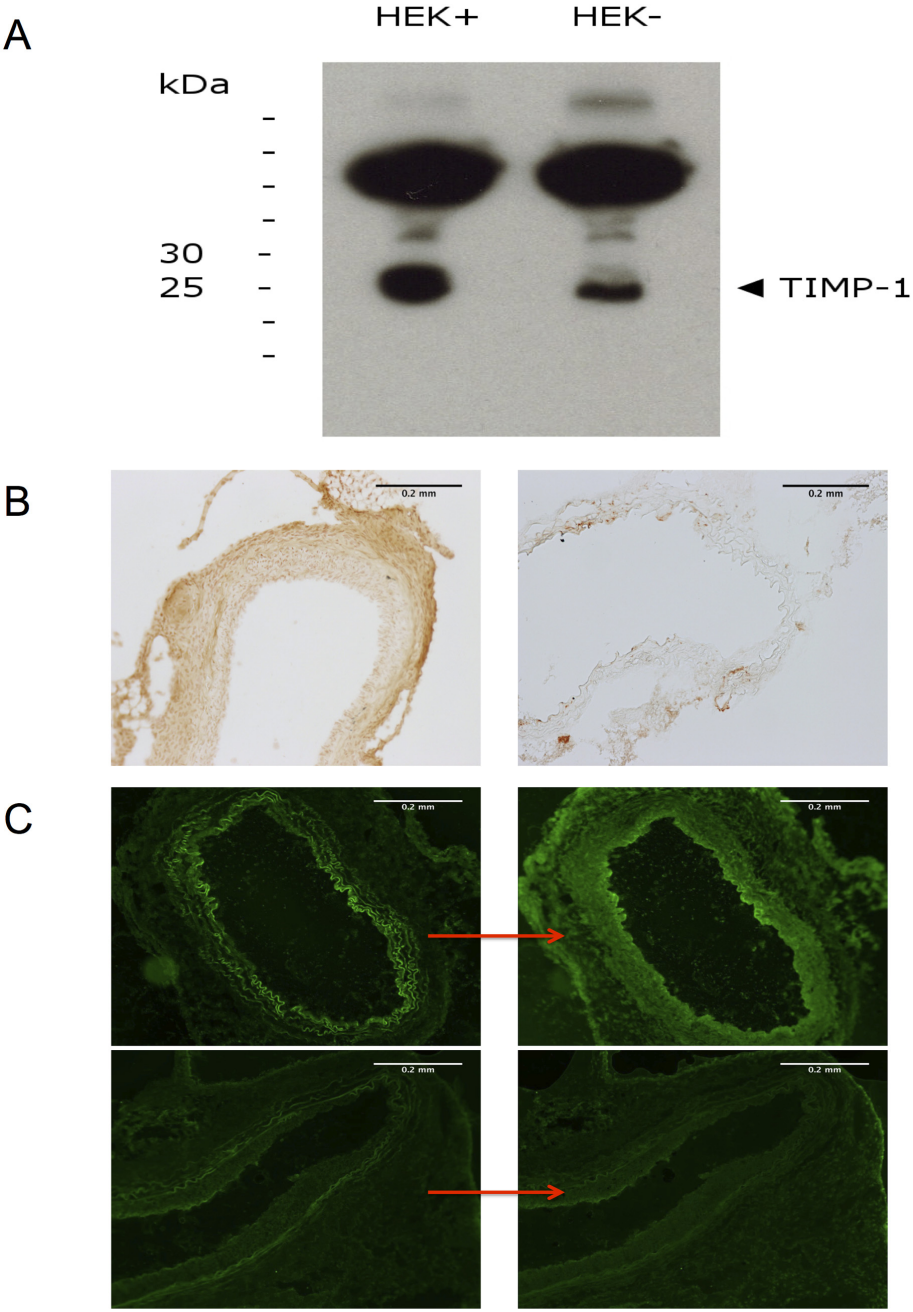
Overexpression of hTIMP-1 after Ad.hTIMP-1 Transduction. A: Western blot analysis for proof of hTIMP-1 overexpression after transdudtion with Ad.hTIMP-1 in HEK-293 cells. Ad.hTIMP-1 transduced cells (HEK+) demonstrated a strong signal for hTIMP-1 above the 25kDa lane (left), whereas untreated native HEK-293 (HEK-) showed weaker lane for hTIMP-1 (right). B: Immunohistochemistry of hTIMP-1 in aortic grafts after transduction with Ad.hTIMP-1 (100x magnification). Even 30 days after transduction sufficient transgene expression is detected in all layers of the aorta (left). Untreated marfan aorta was used as negative control (right). C: *In situ* zymography (100x magnification) of untretead (upper row) and Ad.hTIMP-1 treated mgR/mgR marfan grafts (lower row). Increase of light emission represents conversion of collagen substrate during incubation (24h) by Matrix-Metalloproteinases (left: pre incubation). After Ad.TIMP-1 transduction and incubation (down, right) we saw less MMP activity than in untreated aortas (up, right).

Western blot analysis demonstrated overexpression of hTIMP-1 after adenoviral gene transfer in HEK-293 cells. The hTIMP-1 protein migrated at an apparent molecular weight of 25 to 30kDa. The molecular mass of TIMP-1 ranges vom 28.5–34 kDa according to the degree of glycosylation [23]. Furthermore, immunohistochemical examination of aortic grafts illustrated overexpression of hTIMP-1 30 days after transduction and surgery (Fig 1B). We did not detect hTIMP in untreated Marfan grafts after implementation of the same staining protocol (negative control). The hTIMP-1 transgene was located in every part of the vessel but is concentrated in the intimal and adventitial regions. Collagen *in situ* zymography confirmed successful inhibition of MMP1, -2, -3, -9, and -13 in aortic grafts 30 days after treatment with Ad.hTIMP-1. Qualitatively, the untreated mgR/mgR aorta converted more collagen substrate during incubation than mgR/mgR aorta previously transfected with Ad.h-TIMP-1 (Fig 1C).

### Overexpression of hTIMP-1 Does Not Alter Number of Elastin Breaks Representing the Vascular Phenotype of Marfan Mice

**Fig 2:**
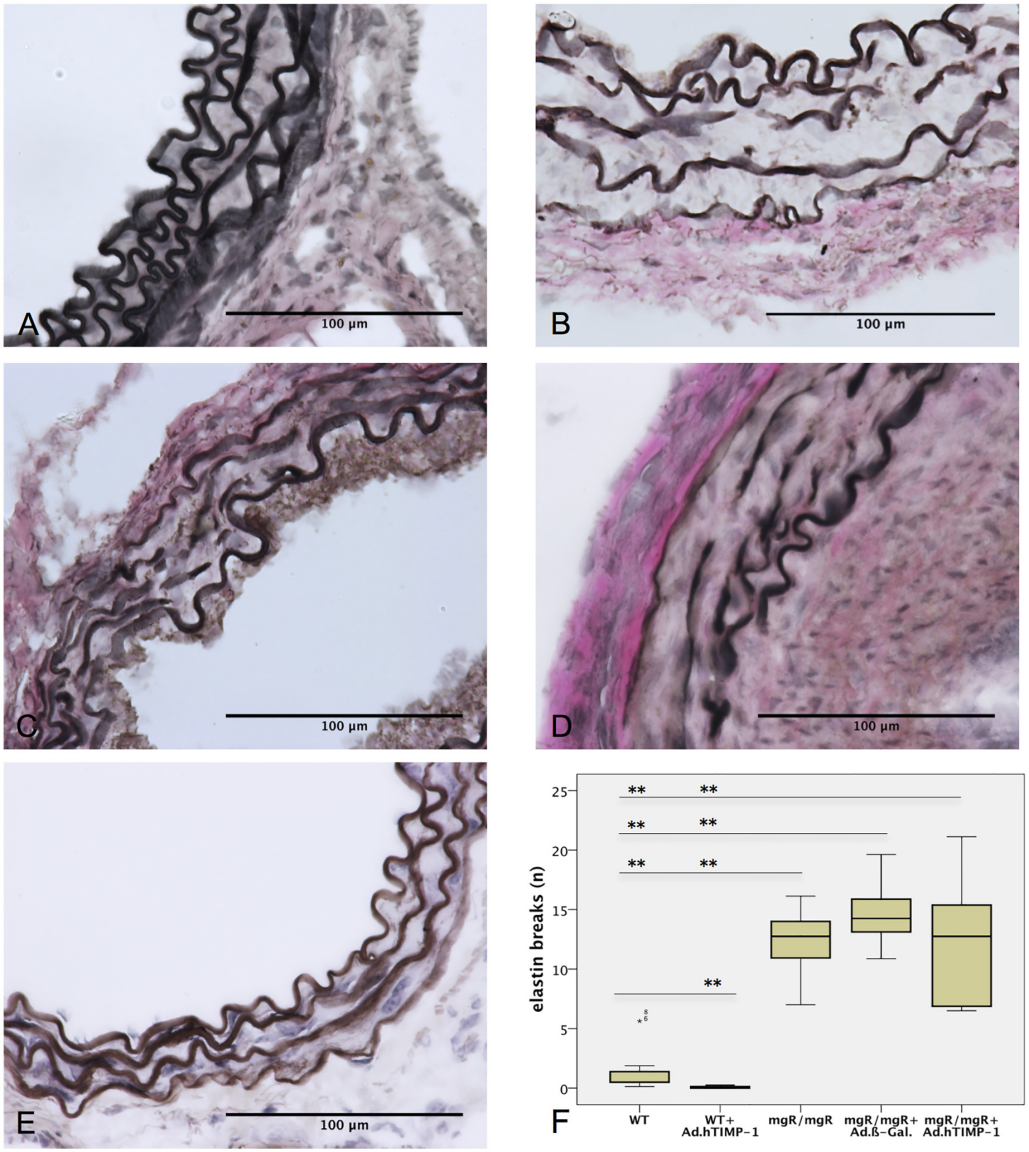
Elastolysis is not Reduced in MgR/mgR Marfan Grafts after Adenoviral Overexpression of hTIMP-1. A-E: Van Gieson stain (400x magnification) of 30-day old mouse aortic grafts (n = 7) after heterotopic infrarenal transplantation. Untreated WT (A) and Ad.hTIMP-1 treated WT (E) show intact elastin layers (1.4 and 0.1 breaks per view field). Native mgR/mgR marfan grafts (B, 12.3 breaks) as well as Ad.β-Gal. (C, 14.7 breaks) and Ad.hTIMP-1 (D, 12.1 breaks) transduced mgR/mgR marfan grafts present severe elastolysis within the aortic media. F: Mean number of elastin breaks within the aortic media (n = 7, counted at 400x magnification): Compared to untreated mgR/mgR and Ad.β-Gal transduced mgR/mgR aortas elastin degradtation was not reduced by andeoviral transduction of Ad.hTIMP-1 (p = 0.902 and p = 0.383). WT, wildtype aorta; mgR/mgR, Marfan aorta; Ad.β-Gal, treated with adenovirus coding for β- galactosidase; Ad.hTIMP-1, treated with adenovirus coding for human TIMP-1. *p-value ≤0.05; ** p-value ≤0.001, calculated using Mann-Whitney-U-Test.

We expected to reduce elastolysis by hTIMP-1 overexpression. The number of elastin breaks within the media was endpoint for quantification of elastolysis (measured in breaks per view field at 400x magnification). As expected untreated WT (Fig 2) and Ad.hTIMP-1 treated WT grafts did not show an increased level of elastin breaks after 30 days (Table 1). The mean quantity of elastin breaks in untreated mgR/mgR grafts was 12.25, which represented significantly more than in native or Ad.TIMP-1-treated WT grafts (p = 0.001 and p = 0.001; Table 2). Surprisingly, Ad.hTIMP-1 treated (12.13 breaks) and Ad-β-Gal.-treated mgR/mgR grafts (14.68 breaks) did not differ statistically in the quantity of elastin breaks (p = 0.383). Therefore, we conclude that overexpression of hTIMP-1 by adenoviral transduction did not reduce elastolysis of Marfan aorta (compared to native mgR/mgR Marfan mice, p = 0.902).

**Table 1:** Number of elastin breaks within the aortic media 30 days after transplantation.

n = 7	WT	Marfan	Marfan + Ad.β-Gal	Marfan + Ad.hTIMP-1	WT + Ad.hTIMP-1
elastin breaks per view field (400x magnification)*	1.43	12.25	14.68	12.12	0.07

WT, wildtype aorta; vs., versus; Marfan, Marfan aorta; Ad.β-Gal, treated with adenovirus coding for β- galactosidase; Ad.hTIMP-1, treated with adenovirus coding for human TIMP-1.

**Table 2:** Statistical evaluation of morphometric analysis.

p-values*			elastin breaks	neointimal Index
**WT**	**vs.**	**WT Ad.hTIMP-1**	0.002	0.534
**WT**	**vs.**	**Marfan**	0.001	0.053
**WT**	**vs.**	**Marfan Ad.hTIMP-1**	0.001	0.001
**WT**	**vs.**	**Marfan Ad.β-Gal**	0.001	0.004
**Marfan Ad.hTIMP-1**	**vs.**	**Marfan**	0.902	0.001
**Marfan Ad.hTIMP-1**	**vs.**	**Marfan Ad.β-Gal**	0.383	0.038
**Marfan Ad.hTIMP-1**	**vs.**	**WT Ad.hTIMP-1**	0.001	0.001
**Marfan**	**vs.**	**WT Ad.hTIMP-1**	0.001	0.017
**Marfan**	**vs.**	**Marfan Ad.β-Gal**	0.165	0.053
**Marfan Ad.β-Gal**	**vs.**	**WT Ad.hTIMP-1**	0.001	0.001

Neointimal index, neointima / (neointima + lumen) in %, WT, wildtype aorta; vs., versus; Marfan, Marfan aorta; Ad.β-Gal, treated with adenovirus coding for β- galactosidase; Ad.hTIMP-1, treated with adenovirus coding for human TIMP-1.

*p-values were calculated using Mann-Whitney-U-Test, p-values ≤0.05 were considered as significant and p-values ≤0.001 as highly significant.

### Aorta of Marfan but Not Wildtype Mice Develop Intimal Hyperplasia after Adenoviral Gene Therapy

**Fig 3:**
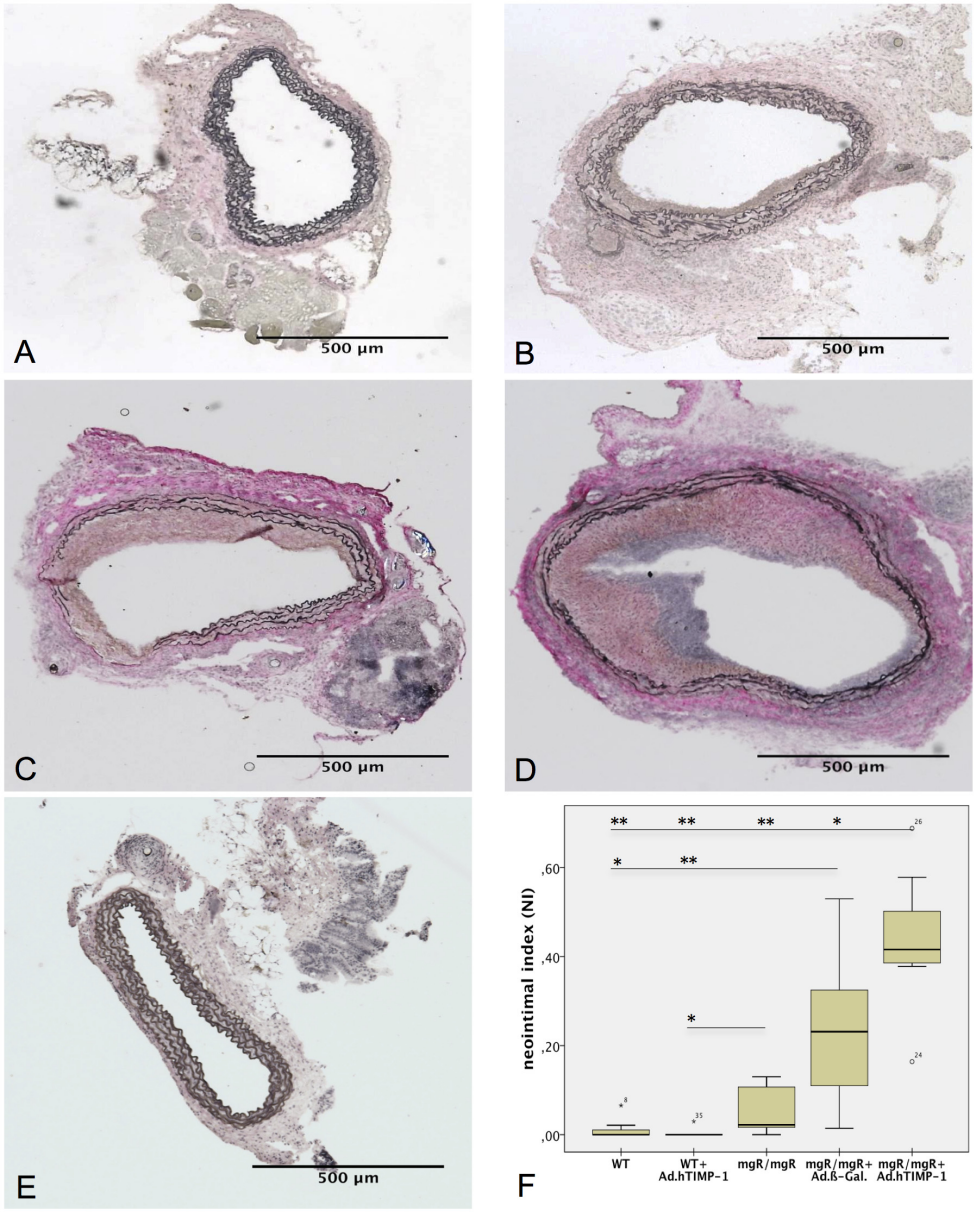
Intimal Hyperplasia in mgR/mgR Marfan Grafts after Adenoviral Transduction. A-E: Van Gieson stain of 30-day old mouse aortic grafts after heterotopic infrarenal transplantation (40x magnification). WT grafts point out almost no (A, NI = 1.24%) and native mgR/mgR Marfan grafts just a minor stenosis of the lumen (NI = 5.7%). Marfan graft after treatment with adenoviral vectors coding for β-galactosidase (C, NI = 23.05%) and hTIMP-1 (D, 43,45%) display intimal hyperplasia narrowing the lumen (p = 0.038). In contrast, wildtype vessels do not present intimal hyperplasia after transduction with Ad.hTIMP-1 (E, NI = 0.00%). F: Mean of neointimal index in 30-day old transplanted aortas (n = 7). Compared to native mgR/mgR grafts NI is significant greater in Ad.hTIMP-1 (p = 0.026) and borderline significant in Ad.β-Gal. transduced mgR/mgR grafts (p = 0.053). WT, wildtype aorta; mgR/mgR, Marfan aorta; Ad.β-Gal, treated with adenovirus coding for β-galactosidase; Ad.hTIMP-1, treated with adenovirus coding for human TIMP-1; NI, neointimal index, neointima / (neointima + lumen). *p-value ≤0.05; ** p-value ≤0.001, calculated using Mann-Whitney-U-Test (n = 7).

The neointimal index was calculated as parameter for intimal hyperplasia (IH), which conceivably could be induced either by the virus or the procedure or both (Fig 3 and Table 3). WT grafts (untreated as well as Ad.hTIMP-1-transduced) did not show prominent stenosis of the lumen (1.24% and 0.00%, p = 0.002, Table 2). Untreated mgR/mgR aortas developed a small stenosis of 5.7% which is statistically larger compared to Ad.hTIMP-1 transduced WT (p = 0.026) but not to native WT aorta (p = 0.053). In contrast Ad.hTIMP-1 and Ad.β-Gal perfused mgR/mgR grafts displayed the severest hyperplasia of the intima resulting in major stenosis of the aortic lumen (43.45% and 23.5%, p = 0.038). Compared to the untreated mgR/mgR Marfan aorta that did not have contact with adenovirus, the NI was significantly larger in Ad.hTIMP-1-treated (p<0.001) and borderline significant in Ad.β-Gal-treated mgR/mgR aorta (p = 0.053).

**Table 3:** Neointimal index as parameter for stenosis within aortic grafts 30 days after transplantation.

n = 7	WT	Marfan	Marfan + Ad. β-Gal	Marfan + Ad.hTIMP-1	WT + Ad.hTIMP-1
Neointimal Index (NI, %)*	1.2	5.7	23.5	43.5	0.0

Neointimal index, neointima / (neointima + lumen) in %; WT, wildtype aorta; Marfan, Marfan aorta; Ad.β-Gal, treated with adenovirus coding for β-galactosidase; Ad.hTIMP-1, treated with adenovirus coding for human TIMP-1.

*arithmetical mean

### Inflammation and Monocyte Invasion after Adenoviral Contact in Marfan Tissue

**Fig 4:**
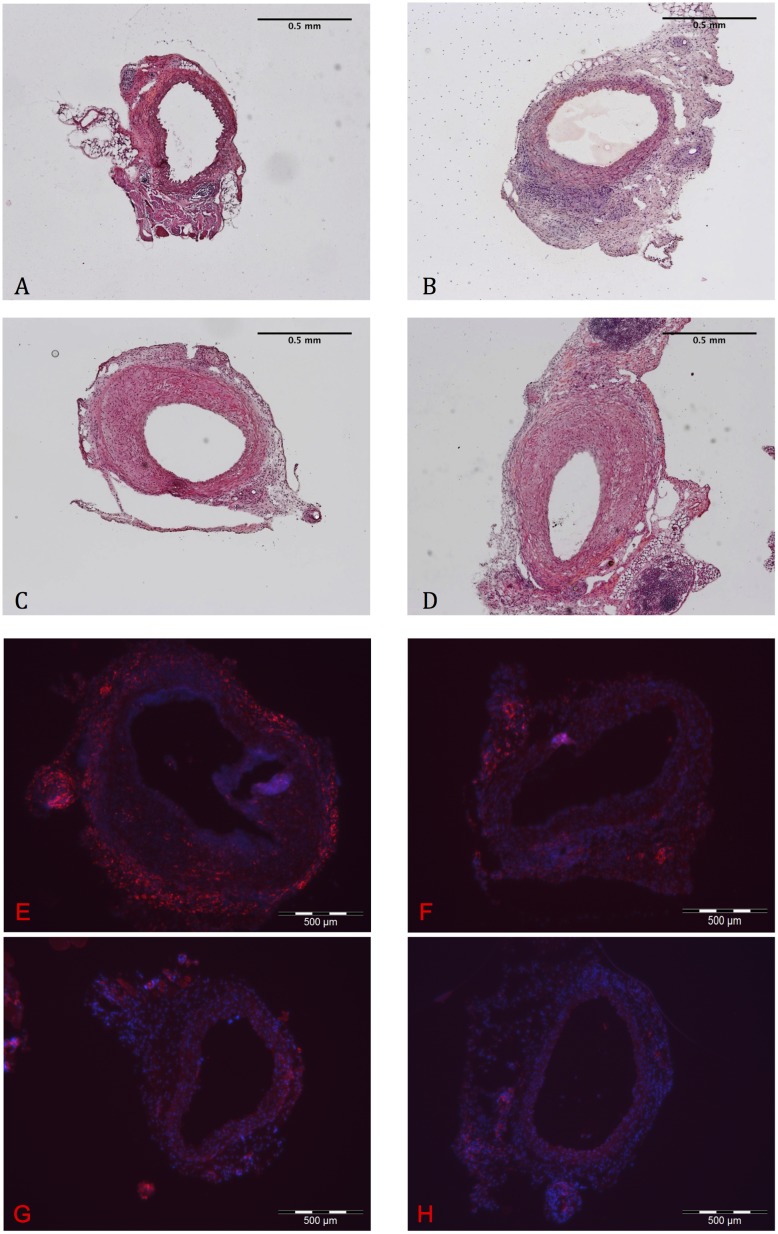
Inflammation in mgR/mgR Marfan grafts. A-D: H&E staining at 40x magnification. Untreated (B) as well as Ad.hTIMP-1 (C) and Ad.β-Gal (D) treated mgR/mgR Marfan vessels present invasion of inflammatory cells (blue). No substantial cellular inflammation is seen in untreated WT grafts (A). E-H: F4/80 immunohistochemical staining (40x magnification) to display monocyte infiltration (red) and counterstain with DAPI to visualize nuclei (blue). Ad.hTIMP- (E) and Ad.β-Gal. transduced mgR/mgR Marfan graft (F) show intense invasion of monocytes within the adventitia and neointima, whereas no relevant amount of monocytes is observed in virus untreated WT (G) and mgR/mgR Marfan aorta (H). WT, wildtype aorta; mgR/mgR, Marfan aorta; Ad.β-Gal, treated with adenovirus coding for β- galactosidase; Ad.hTIMP-1, treated with adenovirus coding for human TIMP-1.

H&E staining of grafts visualized signs of severe inflammation and a large amount of recruited inflammatory cells after adenoviral transduction only in the mgR/mgR aorta, but not in the WT aorta (Fig 4A–4D). In detail we determined the invasion of monocytes by immunohistochemical staining in the aorta of mgR/mgR mice after transduction of adenovirus. (Fig 4E–4H).

### Endothelial Barrier of Marfan Aorta Is Permeable for Large Proteins and Shows Alterations of the Basement Membrane

**Fig 5:**
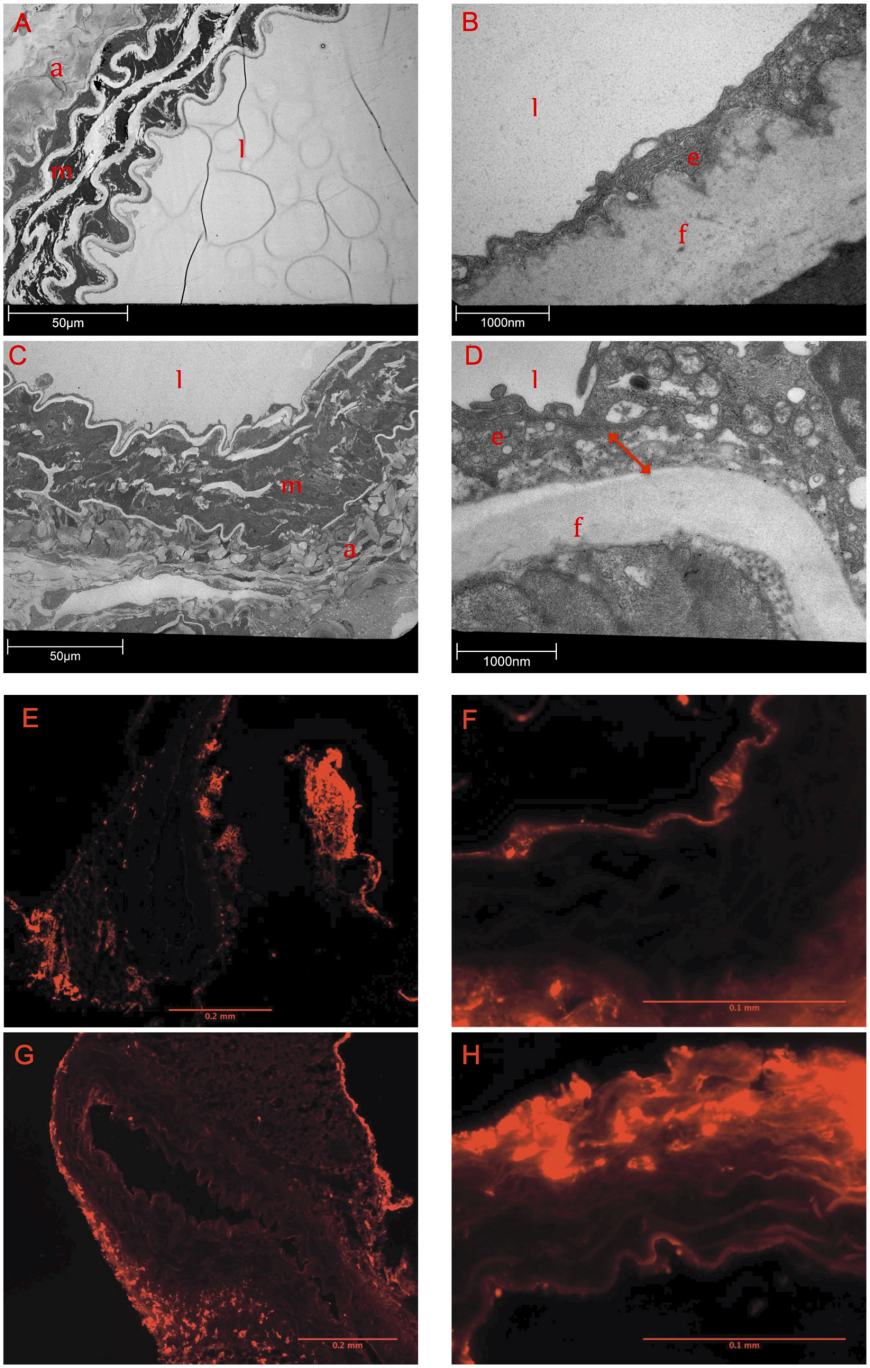
Defect of the Endothelial Barrier in Marfan Tissue. A-D: Analysis of wildtype (WT; A, B) and Marfan aortas (C, D) after transduction of Ad.hTIMP-1 by transmission electron microscope. At 400x magnification characteristic degradation of ECM compounds are found in the Marfan aorta. (C). The integrity of the endothelial-ECM transition is lost in Marfan tissue (D, 16000x magnification) and the basolateral space imposes irregularly wide (D, red arrow) compared to WTaorta (B). Adventitia (a), endothelial cell (e), elastin fiber (f), vessel lumen (l), aortic media (m). E-H: Fluorescence microscopy after perfusion with fluorochrome-labeled bovine albumin. In untreated WT aortas light emission is concentrated at the endothelial layer and the adventitia (E, 100x magnification). Albumin is not able to pass endothelial layer (F, 400x magnification). Native mgR/mgR Marfan aorta displays a significant permeability of the endothelial barrier for albumin (G, 100x; H, 400x). Compared to WT tissue albumin fluorescence (relative brightness) within the media is significant greater in mgR/mgR Marfan tissue (465.38 ± 104.23 vs. 660.20 ± 167.85, p = 0.037, Mann-Whitney-u-Test, n = 6).

Pictures taken by transmission electron microscopy demonstrated substantial differences between the WT and the MFS samples. (Fig 5A–5D): After incubation with Ad.TIMP-1 for 30 minutes mgR/mgR aorta impressed by a very flimsy and bulked basement membrane (BM). The basolateral space was unusually wide and detached. In contrast, the transduced WT aorta presented a common structure of the BM and a compact basolateral space. Based on our TEM results we hypothesized a substantial endothelial dysfunction in native MFS tissue. In order to proof our hypothesis we additionally performed an albumin perfusion test on native, virus-untreated and not transplanted MFS and WT tissue. Incubation with fluorochrome-labeled albumin revealed a substantial defect of the endothelial barrier for large proteins in native, not virus-exposed Marfan tissue: Compared to untreated WT mice aorta, untreated mgR/mgR Marfan aorta showed significant more light emission that correlates with increased albumin diffusion through the endothelial barrier (p = 0.037, Fig 5E–5H, Table 4).

**Table 4:** Endothelial-Diffusion test with fluorochrome labeled Albumin.

n = 6	WT	Marfan	p-value
aortic media—relative brightness*± standard abbreviation	465.38 ± 104.23	660.20 ± 167.85	0.037

WT, untreated and not transplanted wildtype aorta; Marfan, untreated and not transplanted Marfan aorta. Relative brightness was measured at 400x magnification in 9 different parts of the aorta and arithmetical mean was calculated. P-values were calculated using Mann-Whitney-U-Test (n = 6) and p-values ≤0.05 were considered as significant.

*arithmetical mean

## Discussion

Inflammation and degradation of elastin fiber networks by MMPs are known to play a crucial role in the formation of aortic aneurysms in patients with MFS and the mgR/mgR Marfan mouse model [1, 17]. Based on aneurysms caused by structural weakness of the aortic wall, spontaneous dissections or even ruptures are still the major life-threatening complication in MFS [2]. Broad inhibition of MMPs—for instance by overexpression of TIMP-1—therefore may represent a useful approach to reduce MMP induced inflammation and elastolysis within the aortic media. The non-specific MMP inhibitor doxycycline has demonstrated its potential to reduce aortic aneurysm formation in mgR/mgR mice [16]. Overexpression of TIMP-1 in vessels by adenoviral transduction has been performed successfully before in different study models with the approach to inhibit inflammation [24, 25]. Adenoviral gene transfer of hTIMP-1 and, surprisingly, also of MMP-3 resulted in reduced development of intimal hyperplasia in vein grafts [26]. Therefore the concept of MMP-inhibition for reduction of MMP-triggered elastolysis in the murine mgR/mgR aorta seems promising. We are the first to describe adenoviral hTIMP-1 transduction of murine mgR/mgR aorta. In this work we demonstrated sufficient expression of hTIMP-1 even 30 days after adenoviral transduction by immunohistochemical staining. Furthermore, collagen *in situ* zymography revealed efficient inhibition of MMP1, -2, -3, -9 and -13 in aortic grafts by overexpressed hTIMP-1. Therefore, we expected to reduce elastin defragmentation of mgR/mgR aorta by overexpression of hTIMP-1. Surprisingly, we instead observed signs of severe inflammation and hyperplasia of the intima in mgR/mgR aorta after contact with the adenovirus. Elastin breaks within the media of Marfan aorta were not reduced as expected, presumably due to an overshooting immune response. Interestingly, the WT aorta showed no signs of inflammation and lumen narrowing after the same protocol of transgene transduction. In our study adenoviral transfected mgR/mgR mice revealed even higher NI if Ad.hTIMP-1 was applied. We can just assume, but inhibition of MMP seems to play a crucial role in formation of intimal hyperplasia. An ancient study showed reduced neointimal formation in vein grafts after MMP-3 overexpression [26]. Based on that hypothesis overactivity of MMP inhibitors, such as TIMPs, might even provoke the vessel stenosis under special conditions.

In contrast to our findings, overexpression of hTIMP-1 has shown its potential to reduce inflammatory processes and intimal hyperplasia: In an in vitro model overexpression of hTIMP-1 prevents migration of smooth muscle cells, which is known to be one cellular mechanism for intimal hyperplasia [22]. Another study revealed that adenoviral mediated hTIMP-1 decreases intimal hyperplasia in saphenous vein grafts [24]. In contrast, an overshooting immune response (invasion of inflammatory cells, intimal hyperplasia) after application of too high titers of adenovirus was described as well [27]. To avoid the well-described primary toxicity of adenoviral vectors, we applied a protocol with reduced viral titers as described before [26]. Because all animals were generated in the same breed and have an identical genetic background, an immune response against the graft cannot explain our findings. Instead, it seems that the Marfan aorta does not tolerate the same concentration of adenovirus as the WT aorta does. This surprising observation can only be explained by specific characteristics of Marfan tissue on a cellular and molecular level. Our tissue examinations by transmission electron microscope and the albumin diffusion demonstrate functional defects and morphologic alterations of the endothelial cell layer both in virus-exposed and native not virus-treated tissue. After contact to adenovirus the basolateral space is abnormally wide and loose. Additionally, albumin diffusion through the endothelial barrier of virus untreated and not transplanted Marfan aortas is significantly increased compared to the native WT aortas.

A defect of the endothelium is a risk factor for the formation of intimal hyperplasia [28]. Circulating cells are able to pass the defect barrier and to bind to the exposed subendothelial matrix. The pathological mechanism is comparable with the etiology of thromboembolic processes [28–30]. Furthermore, it is known that coagulation disorders in viral infectious diseases, such as Dengue Hemorrhagic Fever, are associated with a defect and dysfunction of the endothelium [31]. However, because of the evident different pathophysiologic mechanisms, we do not think that virus-endothelium interactions are the major pathophysiologic trigger for aneurysm formation in Marfan syndrome. Otherwise we would expect to see aortic atherosclerosis and thromboembolic processes instead of aneurysm formation as major vascular pathology in Marfan syndrome. But in our specific experimental setting with high local virus titers, it is plausible that the development of neointima in mgR/mgR aortas after contact with adenovirus is related to a defect of the endothelium.

The role of endothelial dysfunction in MFS has been discussed before: Wilson et al. described a selective loss of flow mediated endothelium-dependent vasodilation in the brachial artery of Marfan patients [32]. Besides endothelial (dys-) function, the morphological specifics of the Marfan endothelium have not been discussed in the literature yet. However, endothelial hyperpermeability and paracellular flux play a major role in vascular inflammation [33]. Macrophage chemotaxis is part of cellular inflammation in Marfan tissue and is stimulated by fibrillin-fragments [34, 35]. Not only dysfunction but also the here described morphological changes of the endothelial barrier in Marfan aorta might play a crucial role for cellular inflammation that enables macrophages to enter the aortic media where fibrillin-fragments are located. The finding, that indomethacin treatment is capable of reducing elastin degradation in mgR/mgR Marfan mice by inhibition of inflammatory infiltration underlines this explanatory approach [36]. It is necessary to note that the most important receptor for adenovirus-cell interaction, named CAR ("Cocksackie and adenovirus receptor"), is located behind the barrier of epithelial tissue on the basolateral surface. Only a small amount of applied adenoviruses is able to enter the cell on the apical surface [37, 38]. Although this receptor has not been described in Marfan tissue yet, it was found in endothelial cells of rat carotids [39]. Consecutively, a significant defect of the endothelial barrier might allow adenovirus to bind basolateral CAR and induce overshooting inflammation. This represents a coherent explanation why Marfan aorta shows vulnerability against adenovirus and is permeable for large blood standing proteins and inflammatory cells.

In conclusion, we demonstrated overexpression of hTIMP-1 after adenoviral mediated gene transfer in HEK-293 cells *in vitro* and in cells of murine thoracic aorta after heterotopic infrarenal transplantation. However, due to severe intimal hyperplasia it was not possible to show the reduction of elastin degradation within the media of the mgR/mgR aorta. Surprisingly, an overshooting immune response after adenoviral transfection was only observed in the Marfan aorta. Furthermore, the mgR/mgR aorta showed a severe integrity defect of the endothelium and increased permeability for albumin. Therefore, our findings underline the crucial role of the endothelium barrier in the pathogenesis of MFS. Further research is necessary to trace this promising but so far almost neglected approach. A better understanding of the abnormal endothelium in MFS may offer new therapeutic options directed against the vascular phenotype in MFS.
